# Epigenetic master regulators HDAC1 and HDAC5 control pathobiont Enterobacteria colonization in ileal mucosa of Crohn’s disease patients

**DOI:** 10.1080/19490976.2022.2127444

**Published:** 2022-09-29

**Authors:** Mélissa Chervy, Adeline Sivignon, Flavie Dambrine, Anthony Buisson, Pierre Sauvanet, Catherine Godfraind, Matthieu Allez, Lionel Le Bourhis, The Remind Group, Nicolas Barnich, Jérémy Denizot

**Affiliations:** a Université Clermont Auvergne, Inserm U1071, USC-INRAE 2018, Microbes, Intestin, Inflammation et Susceptibilité de l'Hôte (M2iSH), Clermont-Ferrand, France; b Institut Universitaire de Technologie, Génie Biologique, Aubière, France; c Gastroenterology Department, CHU Estaing, Clermont-Ferrand, France; d Surgery and Oncology Digestive Department, CHU Estaing, Clermont-Ferrand, France; e Neuropathology Unit, CHU Gabriel Montpied, Clermont-Ferrand, France; f Gastroenterology Department, Hôpital Saint-Louis - APHP, Paris, France; g Université De Paris, Institut de Recherche Saint-Louis, EMily, INSERM U1160, Paris, France; h Hôpital Saint-Louis, Paris, France

**Keywords:** Histone deacetylases, Adherent-Invasive*E. coli*, Crohn’s disease, histone acetylation, High-fat diet

## Abstract

Adherent-invasive *Escherichia coli* (AIEC), which abnormally colonize the ileal mucosa of Crohn’s disease (CD) patients, are known to contribute to the etiopathogenesis of CD. Molecular mechanisms favoring AIEC ileal colonization have not been completely characterized yet. The aim of this study was to investigate whether epigenetic regulators histone deacetylases (HDAC) expression in intestinal epithelial cells of CD patients regulate Enterobacteria and AIEC encroachment to intestinal mucosa. HDAC were inhibited *in vitro* and in CEABAC10 mice to decipher their involvement in the entry of AIEC within host cells. CD ileal samples from the REMIND cohort were used to study the relationship between HDAC expression level and Enterobacteria/AIEC colonization in patients. Mice were fed a westernized diet and orally challenged with AIEC to determine the impact of diet on HDAC expression. Global level of acetylated histone H3 is higher in patients colonized by AIEC bacteria compared to patients non-colonized by Enterobacteria and HDAC inhibition-mediated H3 hyperacetylation promotes the entry of AIEC bacteria within intestinal epithelial cells. HDAC1 and HDAC5 are central and antagonistic in the regulation of AIEC entry within host cells *in vitro*, in mouse models and in ileal mucosa of CD patients. In mice fed a western-type diet, AIEC infection decreases HDAC1 expression, inducing H3 hyperacetylation to favor their own colonization. CD patients under a western diet are more prone to be colonized by AIEC bacteria as such a diet affects intestinal homeostasis, enables AIEC access to intestinal mucosa where they then manipulate host-epigenome to their advantage.

**Abbreviations:**

AIEC Adherent-Invasive *Escherichia coli*; BSA Bovine serum albumin; CD Crohn’s disease; CEABAC10 Carcinoembryonic antigen bacterial artificial chromosome 10; CEACAM Carcinoembryonic antigen-related cell adhesion molecule; FBS Fetal bovine serum; IBD Inflammatory Bowel Disease; HAT Histone acetyltransferase; HDAC Histone deacetylase; kDa KiloDalton; SAHA Suberoylanilide Hydroxamic Acid; Scr Scramble

## Introduction

Crohn’s disease (CD) is an inflammatory bowel disease (IBD) characterized by chronic inflammatory disorders of the gastrointestinal (GI) tract affecting preferentially the young adult. No curative treatment exists for this debilitating and life-long disease which evolves in a relapsing and remitting manner. Only symptomatic treatments are available to limit the frequency and intensity of the inflammatory flares.^1^ Different factors are involved in the development of the disease such as genetic susceptibilities, environmental factors, and microbiota composition.

The incidence of this disease severely increased over the last few decades, industrialized countries with westernized lifestyle (North America, Europe, Australia …) presenting higher incidences than in in-development countries.^2^ One of the major environmental factor involved in the etiology of CD is the type of diet, and more particularly the consumption of a “western diet” in industrialized countries. This diet, characterized by a high intake of fats, sugar and processed foods and a reduced quantity of fibers, is associated with a higher risk to develop CD.^3–7^ Talking in regards with microbiota, most of patients present a dysbiosis with a reduction of the global microbial diversity, and an increase of the *Proteobacteria* phylum compared to healthy controls.^8–10^ Enterobacteria, members of the *Proteobacteria* phylum can represent up to 100% of the aero-anaerobic microbiota associated with the ileal mucosa in CD and particular pathobiont invasive strains of *Escherichia coli*, designated as the pathotype adherent-invasive *E. coli* (AIEC), have been found in higher prevalence in CD patients compared to healthy controls (21–63% *vs* 0–19%).^11–13^ These pathobiont bacteria can participate in the onset/maintenance of CD by inducing the secretion of pro-inflammatory cytokines and increasing intestinal permeability as demonstrated in genetically susceptible mouse model.^14,15^ In CEABAC10 transgenic mice model mimicking CD, animals fed a high-fat diet (HF diet, enriched in fats) presented dysbiosis with an increase in *E. coli* population as well as increased intestinal permeability. Thus, this diet induced low-grade intestinal inflammation and promoted AIEC intestinal colonization.^16,17^

In addition, CD patients present Paneth cells abnormalities with a reduced expression of defensins.^18–20^ In the work of Alkaissi et al.,^21^ CD patients expressed less human α-defensin 5 (HD5) and AIEC strain LF82 translocation was increased in follicle-associated epithelium (FAE) of CD patients compared to non-IBD controls. Tissue incubation with HD5 reduced AIEC LF82 bacterial passage, demonstrating that defensins are important to regulate AIEC gut colonization. However, considering that some AIEC strains are resistant to these host defense peptides, a better characterization of the molecular mechanisms promoting AIEC gut establishment in CD patients is required to develop specific therapeutic strategies.^22^

Recently, a renewed focus has been carried on the role of epigenetic marks in the pathogenesis of CD. Epigenetic modifications include DNA methylation and histones post-translational modifications (HPTM) which control gene expression through the modulation of the structure of chromatin (formed by DNA and histones proteins) and accessibility of transcription factors to DNA. Most of researches investigated DNA methylation modifications in CD.^23–28^ In contrast, only few studies focused on HPTM in the context of CD.^29^ Among HPTM, histones acetylation, mediated by histones acetyltransferases (HAT/KAT), loosens chromatin structure favoring binding of transcription factors and hence gene expression, whereas deacetylation of histones compacts chromatin limiting gene expression. Histones deacetylases (HDAC) catalyze the removal of acetyl group from histones and non-histones proteins. In humans, 18 HDAC exist divided into two families based on their dependence on specific cofactors and the presence of a conserved deacetylase domain: the histone deacetylase (HDAC) family and the sirtuin family. The deacetylase family includes three classes: class I HDAC (HDAC1, 2, 3, 8), class II HDAC (subclass IIa: HDAC4, 5, 7, 9 and subclass IIb: HDAC6 and 10), and class IV HDAC (HDAC11).^30,31^ HDAC, especially class I HDAC, are crucial in the regulation of intestinal epithelial cell differentiation, and intestinal homeostasis since HDAC1 and HDAC2 intestinal epithelial cells (IEC)-depleted mice present tissue architecture defects with Paneth cell loss, altered barrier function and chronic basal inflammation.^32–35^ HDAC3^ΔIEC^ mice also develop Paneth cell loss as well as impaired IEC functions and dysbiosis.^36^ Furthermore, in two animal models of intestinal inflammation, histone H4 was significantly more acetylated in the inflamed tissue of animals with colitis compared to controls animals.^37^ Also, global acetylation level of histone H4 was significantly increased in inflamed biopsies and Peyer’s patches from CD patients compared to non-inflamed CD patients and controls demonstrating an association between histone acetylation and intestinal inflammation in CD.^37^ Concerning HDAC, a global decrease of the expression of 8 HDAC has been observed in human colonic epithelial cells of CD patients with active disease compared to controls.^38^ These misregulations of HDAC expression could explain the increased acetylation level in histones observed by Tsaprouni *et al*.^37^

Mechanisms leading to an abnormal colonization of the ileal mucosa by Enterobacteria and more specifically by *E. coli* pathobiont bacteria in CD patients have not been completely characterized yet. We hypothesize that in intestinal epithelial cells, there could be a deregulation of the activity or expression of HDAC. Since HDAC are responsible for the deacetylation of histone and non-histone proteins such as transcription factors, their alteration would lead to the misregulation of genes, and some of them might be involved in the control of the entry of AIEC bacteria within cells. Hence, the resulting abnormal genes expression following HDAC alterations could facilitate Enterobacteria and hence AIEC bacteria encroachment to intestinal mucosa. In this work, we show that patients colonized by AIEC bacteria present a higher level of acetylated histone H3 compared to patients non-colonized by AIEC bacteria. Using cellular and animal models, we reveal that HDAC1 expression is central to prevent AIEC colonization of intestinal mucosa and that, in contrast, HDAC5 expression favors AIEC encroachment. These results were confirmed in a large cohort of CD patients, in which we observed imbalanced HDAC1 and HDAC5 expression in AIEC-positive patients. Moreover, HDAC1 expression negatively correlated, whereas HDAC5 positively correlated, with Enterobacteria load associated with ileal mucosa in CD patients. We also observed that AIEC bacteria were able to modulate the host epigenome to their advantage in order to colonize the gut but only in mice fed a high fat diet, which present a specific gut micro-environment. Hence, our results identified HDAC1 and HDAC5 as crucial regulators of pathobiont Enterobacteria colonization of ileal mucosa in CD patients and revealed that targeting HDAC5 could represent an interesting therapeutic strategy to prevent AIEC colonization as well as a food rebalancing.

## Results

### AIEC bacteria are preferentially associated with hyperacetylated histone H3 intestinal epithelium

**Figure 1.**
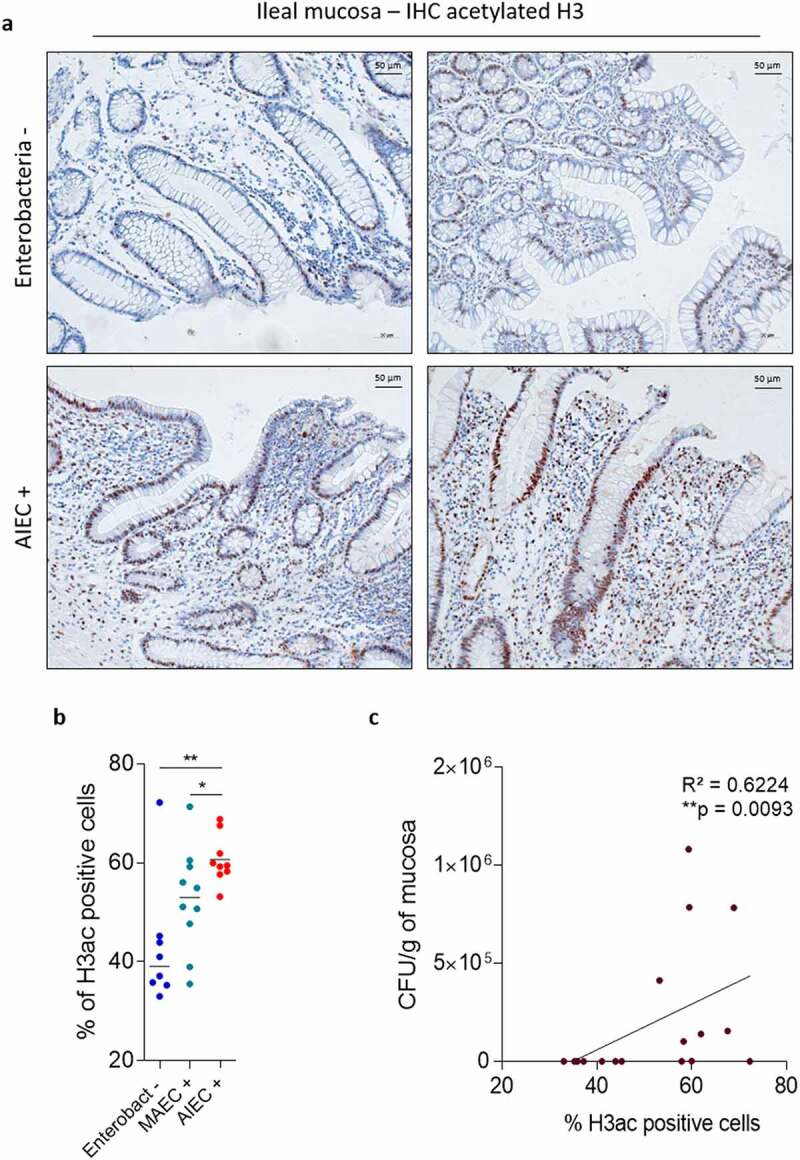
AIEC bacteria are preferentially associated with hyperacetylated histone H3 intestinal epithelium. **a**: Immunohistochemical staining of pan-H3 acetylation mark on mucosa samples from patients non-colonized by Enterobacteria (Enterobacteria -) and colonized by AIEC bacteria (AIEC +). **b**: Percentage of H3ac positive cells in samples of patients non-colonized by Enterobacteria (n = 8), colonized by mucosa-associated *E. coli* (MAEC +, n = 10) or colonized by AIEC bacteria (AIEC +, n = 9). Bars represent medians. Mann-Whitney test, *p < .05, **p < .01. **c**: Correlation between the percentage of H3ac positive cells and Enterobacteria load in patients non-colonized by Enterobacteria (n = 8) and colonized by AIEC bacteria (n = 9). Correlation existing between two variables was assessed by a Spearman test. H3ac: acetylated H3.

The link existing between epigenetic modifications and pathobiont colonization remains unknown. In this context, we aimed to determine whether CD patients colonized by AIEC bacteria presented histone acetylation alterations in intestinal epithelial cells. To this aim, the French multicentric REMIND cohort of CD ileal samples was used (**Table S1**). AIEC bacteria were identified by phenotypical characterization, and Enterobacteria load was quantified from ileal mucosa samples.^39^ The global acetylation level of the histone H3 was studied by immunohistochemical staining using high dilution of primary antibody allowing only the detection of highly acetylated H3 nucleus. H3ac-positive and H3ac-negative epithelial cells were manually counted on mucosa samples from patients which were split into three groups: non-colonized by Enterobacteria (Enterobact -, n = 8), colonized by mucosa-associated *E. coli* (MAEC +, n = 10), and colonized by AIEC bacteria (AIEC +, n = 9). We observed that global H3 acetylation level was increased in MAEC + patients compared to patients non-colonized by Enterobacteria (median Enterobact -: 39.09% *vs* MAEC +: 53.02%). However, this increase did not reach significance. In contrast, we observed a significant increase of global H3 acetylation level in AIEC-carrier patients compared to patients non-colonized by Enterobacteria (median Enterobact -: 39.09% *vs* median AIEC +: 59.45%, p < .01) and compared to patients colonized by MAEC bacteria (median MAEC +: 53.02% *vs* median AIEC +: 59.45%, p < .05). Also, the percentage of H3ac-positive cells positively and significantly correlated with Enterobacteria load associated with the mucosa of patients (**p = .0093), demonstrating that H3 hyperacetylation is associated with AIEC colonization in ileal mucosa of CD patients (Figure 1 and**Figure S1**).

### HDAC inhibition favors the ability of AIEC bacteria to invade IECs in culture

**Figure 2.**
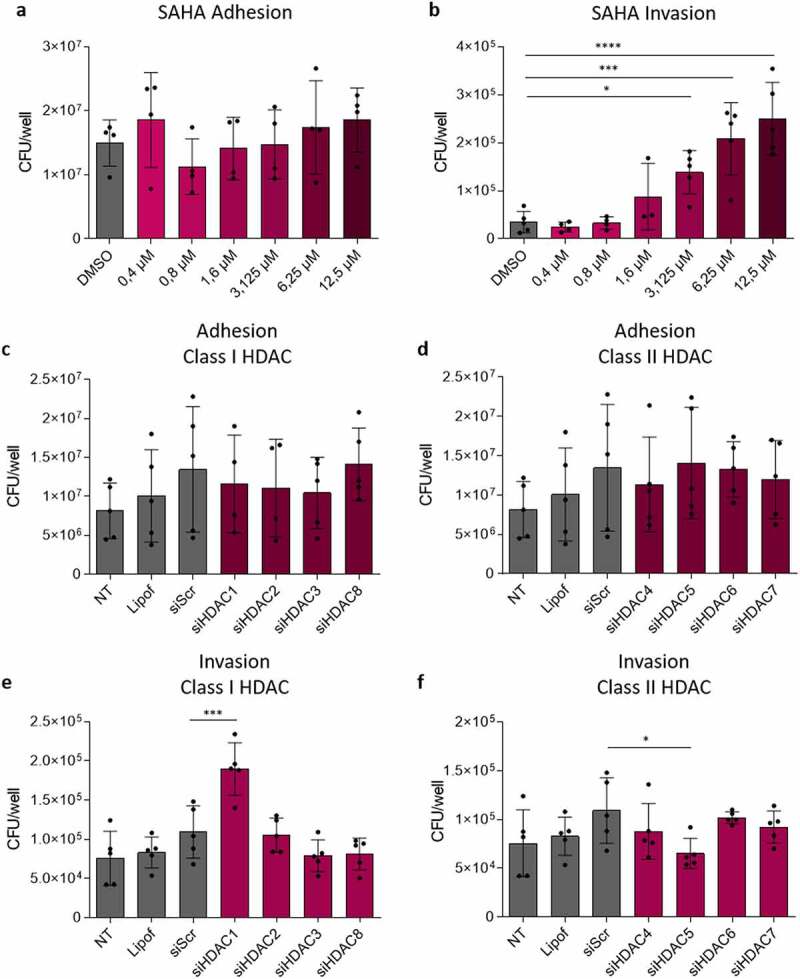
HDAC1 and HDAC5 oppositely control the entry of AIEC bacteria within host cell. **a-b**: Caco-2 cells were treated with increasing concentrations of HDAC inhibitor SAHA for 24 h before infection with the AIEC strain LF82 at MOI 100. Adhesive ability of AIEC strain LF82 was evaluated at 3 h post-infection (a) and a gentamicin protection assay was performed to evaluate invasive ability of the strain in the different conditions (b) (n = 4). **c-f**: Caco-2 cells transfected with control siRNA (siScr) or siRNAs directed against the different HDAC were infected with AIEC strain LF82 at MOI 100. Adherent (c,d) and invasive (e,f) bacteria were numbered at 3 h and 4 h post-infection respectively (n = 5). The results are the mean ± SD. One-way ANOVA, *p < .05, ***p < .001, ****p < .0001. MOI: multiplicity of infection, Lipof: lipofectamine, NT: untreated, Scr: scramble.

As H3 acetylation is mostly regulated by HDAC, we hypothesize that HDAC could be central in the control of AIEC colonization. In order to understand whether HDAC regulate the entry of AIEC bacteria within host cells, Caco-2 IECs were pre-treated with a global HDAC inhibitor (class I and II inhibitor), suberoylanilide hydroxamic acid (SAHA) at different concentrations before infection with the AIEC reference strain LF82. Efficacy and safety of the HDAC inhibitor were confirmed through the observation of a dose-dependent accumulation of H3K9ac mark by western blot and of a cell viability between 78.9% and 95.9%, respectively (**Figure S2A, B**). At 3 h post-infection, adherent bacteria were numbered and no significant differences between conditions were observed, demonstrating that HDAC are not involved in the AIEC adhesion process to IECs (Figure 2a). In contrast, a gentamicin protection assay revealed a significantly enhanced invasion ability of AIEC bacteria in SAHA-pretreated cells compared to DMSO control (vehicle) condition, with a dose-dependent effect (Figure 2b). Similar results were obtained in another cell line (T84) (**Figure S3A-B**). Hence, global HDAC inhibition increases the ability of AIEC bacteria to invade IECs, indicating that a physiological HDAC activity is essential to limit the entry of AIEC pathobiont bacteria within IECs.

### HDAC1 and HDAC5 oppositely control the entry of AIEC bacteria within host cell

SAHA treatment leads to a global HDAC inhibition. Hence, to identify which HDAC are specifically involved in the regulation of invasion process, Caco-2 cells were transfected with siRNAs directed against the different classical HDAC (class I: HDAC1, 2, 3, 8 and class II: HDAC4, 5, 6, 7) to knock-down their expression. Efficacy of siRNAs was ensured by western blot (**Figure S2C**). Cells were infected with AIEC 48 h after transfection, and no difference in the adhesion ability was observed following the knock-down of the different HDAC, confirming that HDAC do not regulate AIEC adhesion to IECs (Figure 2c, d). However, we observed that significantly more bacteria invaded IECs following the silencing of HDAC1 compared to the control condition siScr (mean siScr: 1.09 × 10^5^ CFU/wells *vs* mean siHDAC1: 1.90 × 10^5^ CFU/wells, p < .001) (Figure 2e). In contrast, less invasive AIEC LF82 bacteria were numbered in cells with silenced HDAC5 compared to control condition (mean siScr: 1.09 × 10^5^ CFU/wells *vs* mean siHDAC5: 6.54 × 10^4^ CFU/wells, p < .05) (figure 2f). We obtained equivalent results in T84 cell line (**Figure S3C**). These results demonstrate that HDAC1 and HDAC5 control the entry of AIEC bacteria within host cells but in opposite ways: HDAC1 limits AIEC entry within host cells, whereas HDAC5 promotes the invasion process. Similar results were obtained with three others AIEC strains, suggesting that HDAC-mediated control of AIEC invasion is a common mechanism to AIEC pathobionts (**Figure S4**).

### Class I HDAC inhibition favors AIEC colonization but HDAC5 inhibition limits AIEC colonization in vivo

**Figure 3.**
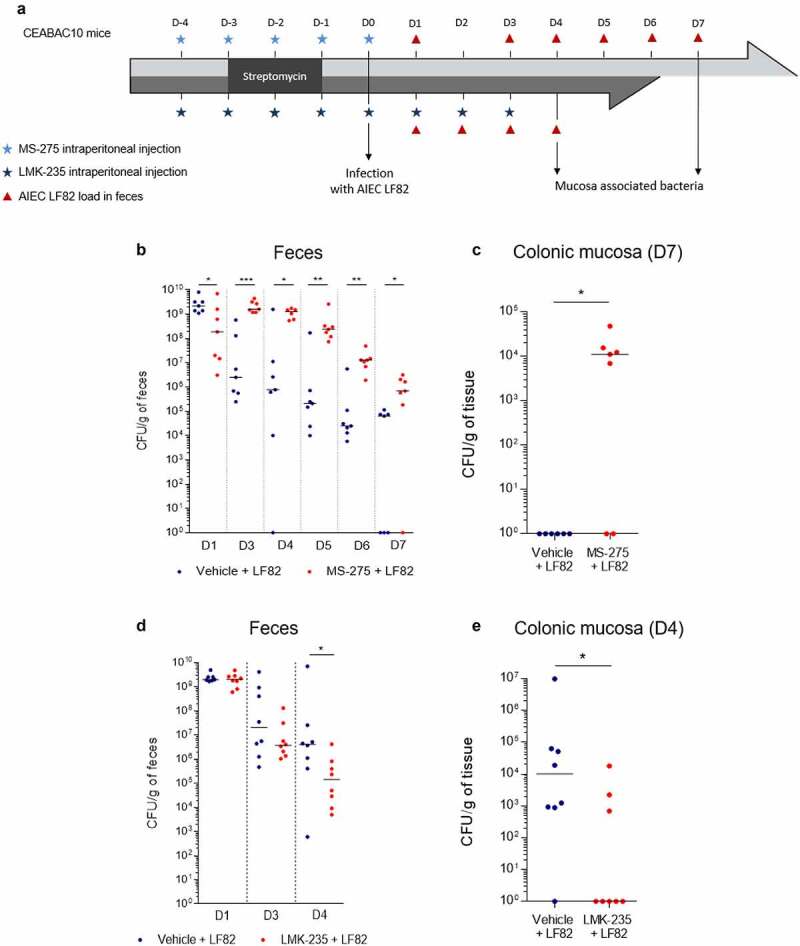
Class I HDAC activity prevents AIEC colonization whereas HDAC4/5 activity favors AIEC colonization *in vivo*. **a**: Experimental protocols of treatment and infection used in the study. **b-e**: AIEC LF82 load in feces at different days post-infection (b, d) and AIEC LF82 bacteria associated with colonic mucosa 7 days (c) or 4 days post-infection (e) were quantified on selective agar plates (n = 7 or 8). Bars represent medians. Mann-Whitney test, *p < .05, **p < .01, ***p < .001.

To confirm our *in vitro* data, CEABAC10 transgenic mice, overexpressing the human *CEACAM6* gene used by AIEC to bind IECs,^40,41^ were daily intraperitoneally injected with MS-275, a class I HDAC inhibitor, during 5 days to inhibit HDAC1. MS-275 treatment efficacy in colonic epithelium was confirmed by analysis of global acetylation level of histone H3 by western blot (**Figure S5**). Mice were then infected with the AIEC strain LF82 and intestinal colonization was followed up (Figure 3a). From day 3 post-infection until the end of the experimentation, significantly more bacteria were present in the stools from MS-275-treated mice compared to feces from control mice receiving only the vehicle of the inhibitor (Figure 3b). Moreover, significantly more AIEC bacteria associated with the colonic mucosa were numbered in MS-275-treated mice compared to control mice for which no AIEC bacteria were associated with the colonic mucosa 7 days post-infection (median Vehicle + LF82: 1.00 CFU/g of tissue *vs* median MS-275 + LF82: 1.11 × 10^4^ CFU/g of tissue, p < .05) (Figure 3c). These results show that MS-275-treated mice are more susceptible to AIEC infection than mice treated with the vehicle. Thus, class I HDAC activity is required to prevent AIEC intestinal colonization *in vivo*.

A similar experiment was conducted in CEABAC10 mice which were daily intraperitoneally injected with LMK-235, a selective inhibitor of HDAC4 and HDAC5, during the whole experiment in order to block HDAC5 activity. Mice were infected after 5 days of treatment with the AIEC strain LF82 and intestinal colonization was studied (Figure 3a). At day 4 post-infection, significantly less AIEC bacteria were present in the feces of LMK-235-treated mice compared to feces of vehicle-treated mice (median Vehicle + LF82: 4.12 × 10^6^ CFU/g of feces *vs* median MS-275 + LF82: 1.44 × 10^5^ CFU/g of feces, p < .05) (Figure 3d). Also, significantly less bacteria associated with the colonic mucosa in LMK-235-treated mice were numbered at day 4 post-infection, compared to control mice (median Vehicle + LF82: 1.01.10^4^ CFU/g of feces *vs* median MS-275 + LF82: 1.00 CFU/g of feces, p < .05) (Figure 3e). These results demonstrate that LMK-235 inhibitor favors AIEC clearance reducing mice susceptibility to AIEC infection. Hence, *in vivo*, HDAC5 activity promotes AIEC intestinal colonization. Therefore, these experiments demonstrate the opposite role of class I HDAC, HDAC1 particularly, and HDAC5 in the regulation of AIEC intestinal colonization with a protective role of HDAC1, and a detrimental part of HDAC5 confirming our observations in cellular model.

### HDAC1 and HDAC5 expression correlate with Enterobacteria load associated with ileal mucosa in CD patients

**Figure 4.**
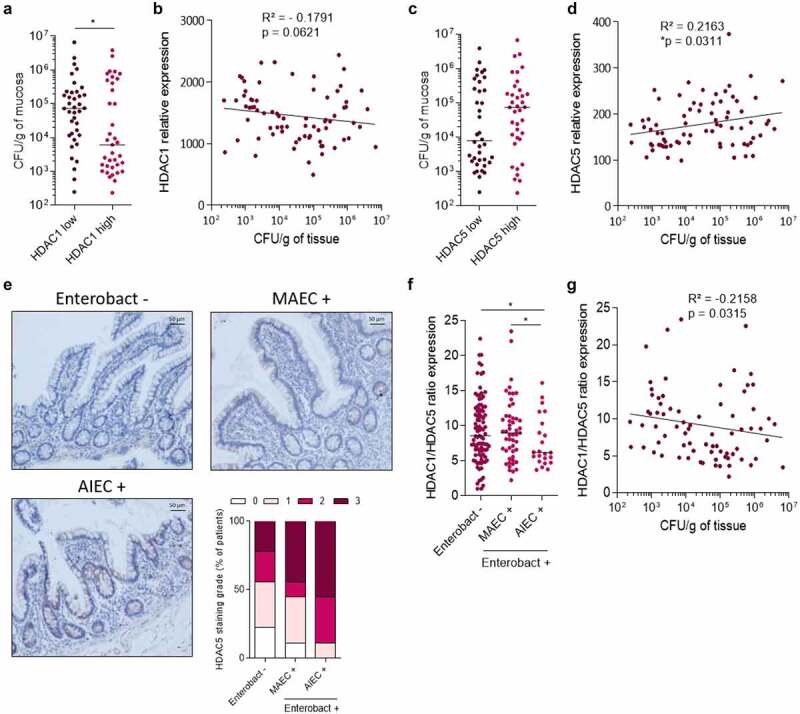
**HDAC1 and HDAC5 expression is correlated with Enterobacteria load associated with ileal mucosa**. HDAC1 and HDAC5 expression levels and Enterobacteria load were quantified in mucosa samples of CD patients from the REMIND cohort. **a**: CD patients’ ileal samples were split into two groups based on HDAC1 expression level (50% in HDAC1 low expression group and 50% in HDAC1 high expression group). The load of Enterobacteria associated with ileal mucosa in each group is plotted on the graph. The results are median. Mann-Whitney test, *p < .05. **b**: Correlation between HDAC1 expression level and Enterobacteria load associated with ileal mucosa. **c**: CD patients’ ileal samples were split into two groups based on HDAC5 expression level (50% in HDAC5 low expression group and 50% in HDAC5 high expression group). The load of Enterobacteria associated with ileal mucosa in each group is plotted on the graph. **d**: Correlation between HDAC5 expression level and Enterobacteria load associated with ileal mucosa. **e**: Immunohistochemical staining of HDAC5 on mucosa samples from patients non-colonized by Enterobacteria (Enterobact -), colonized by mucosa-associated *E. coli* (MAEC +) and colonized by AIEC bacteria (AIEC +). The intensity of the signal in samples was rated as following: 0- no signal, 1- low intensity signal, 2- high intensity signal, 3- very strong intensity signal (n = 9 samples/group) **f**: HDAC1/HDAC5 ratio expression in ileal mucosa of CD patients based on the absence of Enterobacteria associated with the mucosa (Enterobact -), the presence of mucosa-associated *E. coli* (MAEC +) and the presence of AIEC (AIEC +). Bars represent medians. Mann-Whitney test, *p < .05. **g**: Correlation between HDAC1/HDAC5 ratio expression and Enterobacteria load associated with ileal mucosa. Correlation existing between two variables was assessed by a Spearman test (one-tailed).

As we identified the roles of HDAC1 and HDAC5 in the control of the entry of AIEC bacteria within host cells, we then addressed the relationship existing between HDAC1, HDAC5 expression and Enterobacteria in CD patients’ ileal mucosa. To this aim, REMIND cohort transcriptomic analysis was used to determine HDAC1 and HDAC5 expression levels in ileal mucosa^42^ as well as quantification of Enterobacteria load from ileal mucosa samples from each 171 samples (**Table S1** and **Figure S6**). CD patients colonized by Enterobacteria (MAEC + and AIEC +) were split into two groups based on their HDAC1 expression level (50% of patients with high HDAC1 expression and 50% of patients with low HDAC1 expression, the cutoff value used was the median of HDAC1 expression level). Interestingly, we observed that patients having HDAC1 low expression were significantly more colonized by Enterobacteria compared to patients with high HDAC1 expression (median HDAC1 low: 73 846 CFU/g *vs* median HDAC1 high: 6 154 CFU/g, p < .05) (Figure 4a). Moreover, HDAC1 expression negatively correlated with Enterobacteria load associated with inflamed ileal mucosa although the correlation did not reach significance (p = .0621) (Figure 4b). The same analysis was performed using HDAC5 expression levels. We observed an increase in Enterobacteria load in inflamed ileal mucosa of CD patients expressing high level of HDAC5, compared to patients’ mucosa with low HDAC5 expression level (Figure 4c). Interestingly, a statistically significant positive correlation was observed between HDAC5 expression level and Enterobacteria load associated with ileal mucosa (p = .031) (Figure 4d). These results support our *in vitro* data and further confirm that HDAC1 and HDAC5 expression levels are involved in the control of the load of Enterobacteria associated with ileal mucosa in CD patients.

AIEC bacteria were identified by phenotypical characterization in CD patients’ ileal samples from the REMIND cohort and patients were split into three groups: Enterobact -: patients non-colonized by Enterobacteria (n = 95), MAEC +: patients colonized by mucosa-associated *E. coli* (MAEC) (n = 51), AIEC +: patients colonized by AIEC (n = 25). HDAC5 expression level was studied by immunostaining. We observed that the intensity of HDAC5 signal was higher in AIEC + patients compared to MAEC + patients and patients non-colonized by Enterobacteria (Enterobact -: 44.4% of samples with signal intensity ≥2, MAEC +: 55.6% of samples with signal intensity ≥2 *vs* AIEC +: 88.9% of samples with signal intensity ≥2), demonstrating an association between HDAC5 high expression and AIEC colonization (Figure 4e and **Figure S7**). Interestingly, the HDAC1/HDAC5 ratio expression was significantly lower in AIEC-carrier CD patients compared to MAEC+ CD patients and patients non-colonized by Enterobacteria (figure 4f). These observations suggest that low HDAC1 expression and high HDAC5 expression could favor AIEC selection and colonization in CD patients and that imbalance between HDAC1 and HDAC5 expression could predispose CD patients to be colonized by AIEC bacteria. Also, the HDAC1/HDAC5 ratio expression negatively correlated with Enterobacteria load associated with inflamed ileal mucosa (p = .0315), suggesting that HDAC1/HDAC5 ratio expression could be predictive of the Enterobacteria load and AIEC colonization in CD patients (Figure 4g).

### AIEC infection alters host epigenome in HF-fed mice, enhancing AIEC intestinal colonization

**Figure 5.**
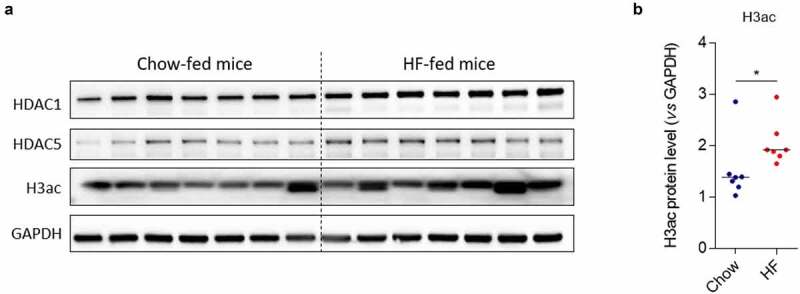
High-fat diet alters histone H3 acetylation in mice. **a**: Western blot targeting HDAC1, HDAC5 expression and H3ac mark in colonic mucosa of mice fed Chow or High-fat (HF) diet. **b**: Quantification of protein expression was performed by assessing band intensities using the Image Lab software (n = 7 mice/per group). Bars represent medians. Mann-Whitney test, *p < .05. H3ac: acetylated H3.

**Figure 6.**
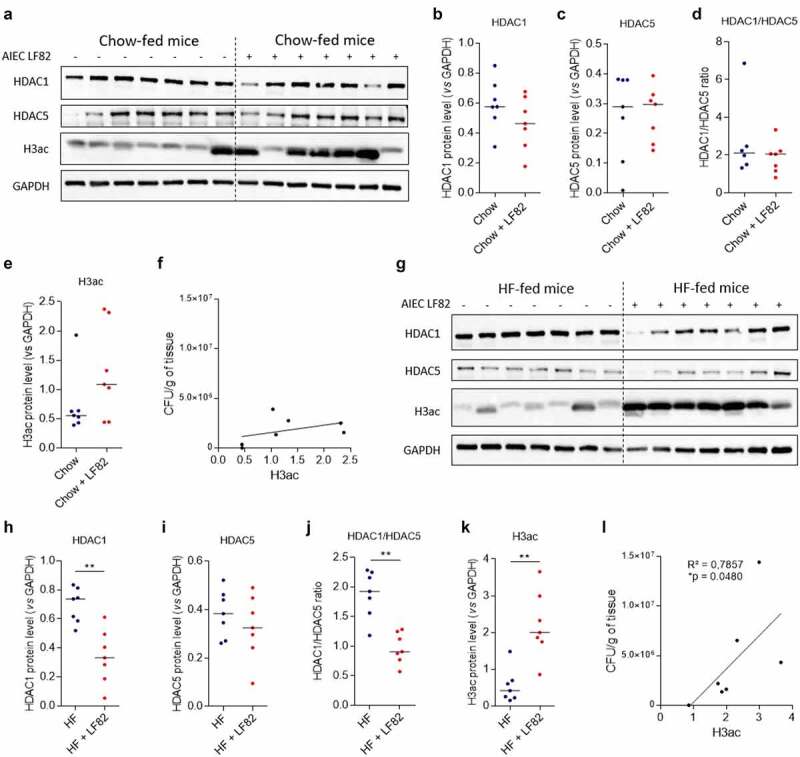
AIEC infection alters host epigenome in HF-fed mice, enhancing AIEC colonization. **a**: Western blot targeting HDAC1, HDAC5 and H3ac expression in colonic mucosa of Chow-fed mice uninfected or infected with AIEC strain LF82. G: HDAC1, HDAC5 and H3ac accumulation in colonic mucosa of HF-fed mice uninfected or infected with AIEC strain LF82 analyzed by western blot. **b-e**, **h-k**: Quantification of protein expression was performed by assessing band intensities using the Image Lab software (n = 7 mice/per group). Bars represent medians. Mann-Whitney test, **p < .01. **f, l**: Correlation between AIEC LF82 load associated with the colonic mucosa of Chow-fed mice (f) or HF-fed mice (l) and global H3 acetylation level. Correlation existing between two variables was assessed by a Spearman test. H3ac: acetylated H3.

So far, we demonstrated that the levels expression of HDAC1 and HDAC5 control the ability of pathobiont Enterobacteria to interact with the intestinal mucosa. However, we now need to understand the causes of the disequilibrium of HDAC expression in CD patients. It is known that the consumption of a diet enriched in fats, sugar with a reduced proportion of fibers is an environmental factor associated with a higher risk to develop CD.^3–7^ We hypothesize that diet could be responsible for the deregulation of HDAC1 and HDAC5 expression. Hence, we fed mice with a high-fat (HF) diet enriched in fats during 4 weeks and studied HDAC1 and HDAC5 expression as well as the global acetylation level of the histone H3 by western blot. A light but significant increase in global acetylation level of H3 in colonic mucosa of HF-fed mice compared to mice fed a standard diet (Chow diet) was observed (median Chow: 1.381 *vs* median HF: 1.918, p < .05), whereas no significant change in HDAC expression was noticed between the two groups of mice (Figure 5). Since HF diet alters the intestinal barrier function allowing bacteria to reach the epithelium,^16,17^ we assessed the effect of a HF diet on the ability of AIEC bacteria to modulate histone acetylation *in vivo*. The infection with AIEC bacteria did neither induce significant modulations of HDAC1 and HDAC5 expression nor modification of H3 global acetylation in colonic mucosa of Chow-fed mice (Figure 6a-f). In contrast, in colonic mucosa of HF-fed mice, AIEC bacteria infection led to a significant decrease in HDAC1 expression (median HF: 0.738 *vs* median HF + LF82: 0.332, p < .01) and to a significantly reduced HDAC1/HDAC5 ratio (median HF: 1.925 *vs* median HF + LF82: 0.904, p < .01), associated with a significant H3 hyperacetylation, which are the conditions we identified as promoting AIEC gut colonization (Figure 6g-k). Interestingly, we observed a positive correlation between H3 acetylation level and the load of AIEC bacteria associated with the colonic mucosa of HF-fed mice, similar to what we observed in CD patients (Figure 6l). Hence, these results indicate that HF diet creates a specific micro-environment in the gut allowing AIEC bacteria to reach intestinal epithelial cells and to modulate the host epigenome to its advantage, enabling invasion of host cells. We then confirmed the requirement of a HF-altered gut environment to allow AIEC-induced epigenetic alterations. Under infection with AIEC bacteria, a significant decrease in HDAC1 expression (median Chow + LF82: 0.759 *vs* median HF + LF82: 0.456, p < .05), an increase in HDAC5 expression as well as a significantly reduced HDAC1/HDAC5 ratio (median Chow + LF82: 6.016 *vs* median HF + LF82: 2.682, p < .01) associated with H3 hyperacetylation were observed in colonic mucosa of HF-fed mice compared to Chow-fed mice (**Figure S8**).

## Discussion

CD patients are abnormally colonized by AIEC bacteria in ileal mucosa but the molecular mechanisms responsible for this increased susceptibility to carry AIEC bacteria are incompletely characterized. In this study, we first observed that H3 histone was more acetylated in patients colonized by AIEC bacteria compared to patients non-colonized by Enterobacteria or by MAEC bacteria. This observation led us to focus our study on the role of acetylation epigenetic regulators, such as histone deacetylases or HDAC, to determine whether these players could have a role in the susceptibility of IECs to be colonized by Enterobacteria and more specifically by AIEC pathobiont bacteria. We observed that global HDAC inhibition favored the entry of AIEC within intestinal cells, demonstrating that HDAC activity limits the AIEC invasion process within host cells. In the literature, Eskandrian et al^33^ demonstrated that HeLa cells treated with a global HDAC inhibitor, Trichostatin A, were more prone to be infected by the invasive bacteria *L. monocytogenes* than untreated cells,^43^ suggesting that regulation of infection by HDAC could be a common mechanism to invasive bacteria. In the work of Mombelli et al,^44^ global HDAC inhibition altered antibacterial defense of macrophages by reducing the phagocytosis and the killing of *Escherichia coli* and *Staphylococcus aureus* by mouse bone marrow-derived macrophages. Moreover, HDAC inhibition resulted in a decreased bacteria-induced production of reactive oxygen and nitrogen species by macrophages. Similarly, Corrêa et al^45^ showed that short-chain fatty acids and SAHA/MS-275 treatment altered phagocytic and killing bacteria capacities of neutrophils in mice infected with a pathogenic bacteria. These results strengthen the crucial role of HDAC in the control of infection by pathogens in different cell types.

The silencing of the different HDAC showed that two HDAC in particular, HDAC1 and HDAC5, regulate bacterial invasion process: while HDAC1 limits the AIEC entry within host cells, HDAC5 favors it. In the REMIND cohort of CD patients, we observed that in inflamed ileal mucosa, patients with low level of HDAC1 and high level of HDAC5 were more colonized by Enterobacteria and carried AIEC. Interestingly, a lower HDAC1/HDAC5 ratio expression was associated with the presence of AIEC in the subgroup of Enterobacteria-colonized patients. Since no transgenic mouse model mimicking AIEC ileal colonization are currently available, the transgenic CEABAC10 mice model, which reproduces the AIEC colonic colonization, was used in this study. In this *in vivo* model, we demonstrated that mice treated with MS-275 were more prone to AIEC infection compared to untreated mice, whereas LMK-235-treated mice were less susceptible to infection compared to control mice confirming the role of class I HDAC and HDAC5 in AIEC intestinal colonization. Hence, it appears that HDAC1/HDAC5 balance expression regulates host susceptibility to be colonized by Enterobacteria. Through promotion of intestinal inflammation, Enterobacteria colonization enhances the risk to select AIEC bacteria, the presence of these pathobiont bacteria associated with the patients’ ileal mucosa increasing the risk of relapse 6-month post-surgery as recently demonstrated by Buisson et al.^39,46,47^

Based on our data, two potential therapeutic strategies to limit Enterobacteria, and more specifically AIEC bacteria colonization could be to (1) activate HDAC1 expression/activity in intestinal epithelial cells or to (2) limit HDAC5 activity. However, the latter strategy seems more realistic than the former, as specific HDAC5 inhibitors have already been developed and used in different studies. Indeed, specific targeting of HDAC5 with HDAC5 silencing or a specific HDAC5 inhibitor (LMK-235) was used in an *in vivo* sepsis model and it resulted in improved mice survival and intestinal permeability, and in a reduced intestinal dysfunction.^48^ These data strengthen the hypothesis that HDAC5 inhibition could represent an interesting therapeutic alternative in the treatment of intestinal diseases. Short-chain fatty acids (SCFA), mainly acetate, propionate and butyrate, are known microbiota-derived HDAC inhibitors and play an important role in the regulation of intestinal inflammation.^49^ However, the molecular mechanism leading to the protective effect of butyrate is not completely understood yet. It was demonstrated that butyrate induces disruption of the subcellular nuclear localization of HDAC5 while in another study, it was shown that butyrate attenuates angiotensin II–induced cardiac hypertrophy I in rats through the inhibition of HDAC5.^50,51^ Hence, these studies clearly highlighted that butyrate can have an effect on HDAC5 activity and suggest that the beneficial effect of butyrate in IBD could be mediated through HDAC5 inhibition. It is also important to keep in mind that HDAC are global regulators of genes expression and modulating such proteins activities could eventually lead to uncontrolled adverse effects. Therefore, it appears important to perform transcriptomic analysis to try to identify specific genes which could be involved in the control of AIEC entry within host cells in order to better understand the link between HDAC and AIEC colonization and to discover new potential target genes.

The higher incidence of CD in industrialized countries, where people are more exposed to smoking, westernized diet, air pollution or chronic stress, than in in-development countries directed us to consider that some environmental factor could alter HDAC1/HDAC5 expression. We focused the study on the effect of the consumption of a western diet enriched in fats and sugar due to its association with a higher risk to develop CD and its detrimental effect on the intestinal barrier function and microbiota promoting AIEC gut colonization.^16^ We demonstrated that mice-fed a HF diet presented an increase in global H3 acetylation level although no significant change in HDAC1 and HDAC5 expression was observed. This increased acetylation level can be the result of a decreased expression of others HDAC or the consequence of a reduced HDAC activity in HF-fed mice undetectable by western blot. AIEC infection resulted in a decreased HDAC1/HDAC5 ratio associated with H3 hyperacetylation only in mice fed a HF diet. These misregulations were not observed in Chow-fed mice. Hence, consumption of a western diet impairs the gut homeostasis, creating a specific intestinal environment. This enables AIEC bacteria to reach the intestinal epithelium where they can alter the expression of epigenetic master regulators HDAC1 and HDAC5 to promote their entry within the host and to favor their colonization.

**Figure 7.**
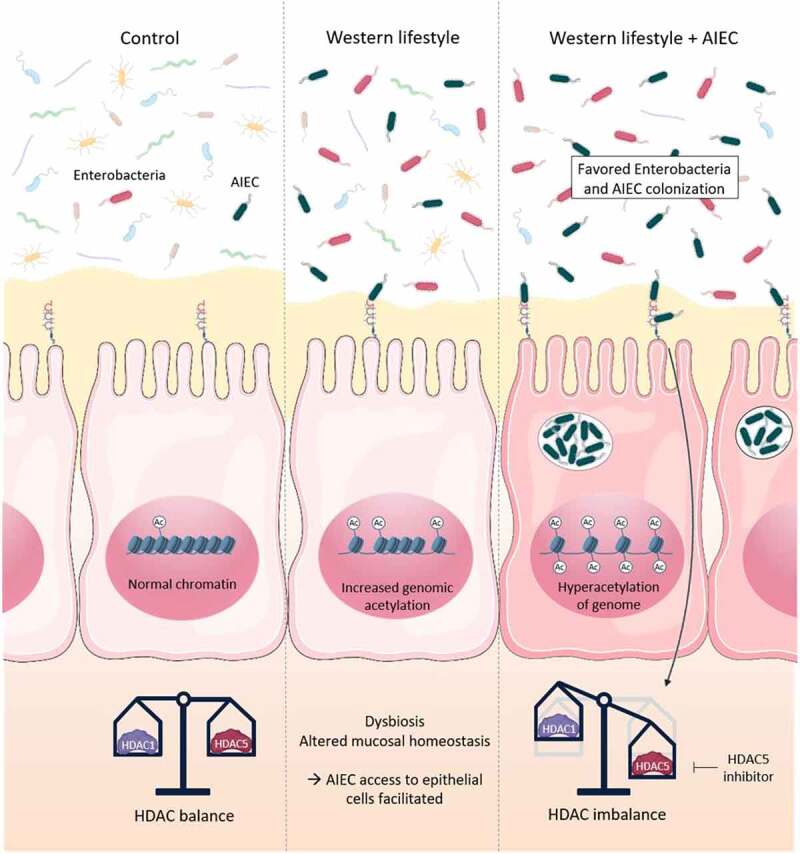
HDAC1 and HDAC5 control Enterobacteria colonization in ileal mucosa of CD patients. The HDAC1/HDAC5 expression balance is crucial in the control of the interaction between Enterobacteria and intestinal epithelial cells in ileal mucosa of CD patients, a reduced HDAC1 expression associated with an increased expression of HDAC5 being conditions favoring Enterobacteria and AIEC gut colonization. Consumption of a high-fat diet leads to alterations of global H3 acetylation level and creates a specific micro-environment that allows AIEC bacteria to reach intestinal epithelium. In this environment, AIEC bacteria are able to imbalance HDAC1 and HDAC5 expressions to their advantage to colonize the gut mucosa. Targeting HDAC5 could represent an interesting approach to prevent AIEC colonization in CD patients. Ac: acetylation.

In this work, we highlighted the central role of chromatin global regulators, HDAC, in the interaction between Enterobacteria and intestinal epithelial cells in ileal mucosa of CD patients. Indeed, the balance between HDAC1 and HDAC5 expression levels controls the ability of pathobiont Enterobacteria to interact with intestinal mucosa. We also demonstrated that HF diet creates a specific micro-environment within the gut which favors AIEC access to the epithelium where they can modulate the host epigenome to their advantage in order to colonize (Figure 7). With these results, a food rebalancing toward a Mediterranean diet, which includes high-fiber foods associated with a reduced consumption of fats and sugar, could limit AIEC gut colonization by restricting intestinal deregulations promoting AIEC access to the epithelium and subsequent AIEC-induced HDAC imbalance. We could also imagine combining HDAC5 targeting strategies with this healthier diet in order to increase the chances to limit Enterobacteria and AIEC colonization of the ileal mucosa in CD patients and prevent relapses.

## Materials and methods

### In vitro experiments

#### Intestinal epithelial cells culture and inhibitor treatment

Intestinal epithelial cells Caco-2, TC7 clone, were cultivated in DMEM medium supplemented with 20% heat-inactivated fetal calf serum (FCS, Dutscher), 2 mM of glutamine (Gibco), vitamins (1%, Dutscher), amino acids (1%, Fisher) and antibiotics cocktail (penicillin, streptomycin, Amphotericin B, 1%, HyClone). Intestinal epithelial cells T84 were cultivated in DMEM/F12 medium supplemented with 10% heat-inactivated fetal calf serum (FCS, Dutscher), 2 mM of glutamine (Gibco), vitamins (1%, Dutscher), 10 mM of Hepes (Dutscher) and antibiotics cocktail (penicillin, streptomycin, Amphotericin B, 1%, HyClone). Cells were seeded into 24-well culture plates at a density of 1.10^5^ cells/well (Caco-2) or 4.10^5^ cells/well (T84) in a medium depleted of antibiotics and were incubated at 37°C in a humidified 5% CO_2_ atmosphere for 24 h. Cells were treated with suberoylanilide hydroxamic acid (SAHA, Sigma), a global HDAC inhibitor, prepared in DMSO at different doses (0.4/0.8/1.6/3.125/6.25/12.5 µM) during 24 h.

#### Viability assay

SAHA cytotoxicity was assessed with a XTT Cell Viability Kit (30007, Biotium®) after a 24 h-treatment according to the manufacturer’s instructions. Briefly, 100 µL of activated XTT solution were added on each well. After 5 h of incubation, absorbance was measured at 450 nm and 630 nm.

#### siRNA transfection

Cells were seeded into 24-well culture plates at a density of 3.5 × 10^4^ cells/well (Caco-2) or 2.10^5^ cells/well (T84) in a medium depleted of antibiotics 24 h before transfection. For each condition, siRNA and Lipofectamine® 2000 (Caco-2) or RNAiMAX (T84) (Invitrogen) were separately diluted in Opti-MEM Reduced Serum Media (Gibco) and incubated 5 min at room temperature before mixing (V/V, final concentration = 10 nM). After 20 min of incubation at room temperature, medium in each well was replaced with 200 µL of the mix and cells were incubated during 6 h at 37°C/CO_2_ 5%. Transfection was stopped by addition of 800 µL/well of medium and cells were incubated 48 h at 37°C/CO_2_ 5%.

#### Bacterial strains, adhesion-invasion assays on IECs

AIEC strain LF82 used for all the experiments was isolated from a chronic ileal lesion of a patient with CD^11^ as well as the AIEC strains CEA614S, CEA618U and CEA212U.^52^ The day of infection, cells were washed twice with PBS before infection with the AIEC strains at a multiplicity of infection (MOI) of 100 bacteria per cell. At 3 h post-infection, cells were washed twice with PBS and lysed with Triton 1X and serial dilutions of the lysates were plated on LB gelosis to number adhesive bacteria. For invasion assay, a gentamicin protection assay was performed to number invasive bacteria. Briefly, medium was replaced at 3 h post-infection with medium supplemented with gentamicin (100 µg/mL) to kill extracellular bacteria. After 1 h of incubation, cells were lysed and invasive bacteria were numbered after dilution and plating of the lysates on LB gelosis.

### In vivo experiments

#### Mice treatments

FVB/N female wild-type (WT) and CEABAC10 transgenic mice^40^ were maintained in our animal care facilities at the University Clermont Auvergne (Clermont-Ferrand, France).

CEABAC10 mice were daily intraperitoneally-injected with MS-275 (5 months, 20 mg/kg, Selleckchem) during 5 days (n = 7) or with LMK-235 (6 weeks, 6 mg/kg, Sigma) during 8 days (n = 8). Mice used as controls only received the vehicle of the inhibitor (MS-275: 4% DMSO, 30% PEG300, distilled water (n = 7); LMK-235: 3.2% DMSO, 30% PEG300, 5% Tween 80, distilled water, n = 8) (Figure 3a).

WT mice (8 weeks) were fed either a standard diet (Chow diet, A04, SAFE®) or a high-fat diet (HF diet) enriched in fats (U8957 Version 1, SAFE®) during 4 weeks. At the end of the 4 weeks, one half of mice were sacrificed and colon was collected.

All infected mice received either the inhibitor or the vehicle, the broad-spectrum antibiotic streptomycin in drinking water (2.5 g/L) during 2 days to disrupt normal resident bacteria of intestinal microbiota and were orally challenged 24 h later with 1.10^9^ AIEC LF82 bacteria cultured overnight in LB medium. Mice were sacrificed 7 (MS-275), 4 (LMK-235) or 8 (HF diet) days after infection.

#### Follow-up of intestinal colonization by AIEC

AIEC LF82 in stools were counted regularly post-infection by homogenization in PBS and numeration on selective agar plates containing Ampicillin (100 µg/mL) and Erythromycin (20 µg/mL). The day of sacrifice, colons were collected and washed in PBS. One centimeter was homogenized in 1 mL of PBS and serial dilutions were plated on selective agar plates to count mucosa-associated bacteria. Results are expressed in colony forming unit (CFU)/g of feces or CFU/g of tissue.

### Analysis on CD patients: the REMIND cohort

#### Description of the REMIND cohort

Patients data are from a multicenter prospective study (9 centers) conducted by the REMIND group (REcherche sur les Maladies INflammatoires Digestives) which aimed to identify predictors of early post-operative endoscopic recurrence.^53^ Briefly, CD’s patients older than 18 years old, presenting ileal or ileocolonic CD and undergoing CD-related ileocolonic resection in absence of intestinal dysplasia or cancer were enrolled (**Table S1**). Mucosal samples were collected from the ileal side of the surgical specimen in the macroscopically inflamed area. All samples were stored at −80°C, at Nice Hospital Biobank (BB-0033-00025), University Côte d’Azur, France.

#### Numbering of E. coli associated with ileal mucosa of CD patients

Samples collected during surgery were washed in phosphate-buffered saline (PBS), crushed (Ultra-Turrax, IKA) and incubated for 15 minutes on a tube rotator at room temperature in the presence of Triton 0.1X. Serial dilutions were then plated on the CPS differential medium (on which *E. coli* is pink, *Klebsiella* blue) or on selective Drigalski agar medium to quantify all the cultivable Gram-negative bacilli (mainly Enterobacteria). Results are expressed in CFU/g of ileal mucosa. *E. coli* identification was validated by mass spectrometry.

#### Identification of AIEC bacteria in ileal biopsies of CD patients

First, a pre-screening test was conducted on 45 *E. coli* strains previously isolated from ileal biopsies. After mixing, the global abilities of these strains to invade intestine-407 epithelial cells (I-407, ATCC) were assessed. Briefly, a maximum of 45 strains per sample were equitably and extemporaneously mixed prior infection of I-407 cells at a MOI of 100 bacteria/cell. After 3 h of infection, a 1 h- treatment with gentamicin (100 µg/mL) was performed to kill extracellular bacteria. Cells were then lysed using Triton 1X and internalized *E. coli* of each mixture were numbered on agar plates. The *E. coli* K-12 strain (non-AIEC strain) was used as negative control whereas the AIEC reference strain LF82 served as positive control. AIEC bacteria from samples with an invasion rate greater than 0.1% of the original inoculum were characterized. AIEC characterization consisted in the analysis of the abilities of at least 4 *E. coli* strains per patients to adhere to and to invade intestinal epithelial cells as well as to survive and replicate within macrophages. Hence, gentamicin protection assays were conducted on I-407 epithelial cell line and THP-1 macrophages (ATCC) as previously described.^11^

#### Immunohistochemical staining

Mucosal samples were embedded into paraffin and 5 µm sections were cut with a microtome. After unwaxing and rehydratation of tissues, antigens retrieval was performed with citrate buffer (10 mM, pH 6). Endogenous peroxidases were blocked in PBS with H_2_O_2_ 0.3% during 30 min and unspecific sites were blocked using PBS with BSA 1% for 1 h. Polyclonal rabbit pan-H3ac antibody (1/1000, #61637, Active Motif) or HDAC5 antibody (1/500, #sc-133106, Santa Cruz Biotechnology) were diluted in PBS with BSA 0.1% and incubated overnight at room temperature. Tissues were incubated 1 h at room temperature with a biotinylated secondary antibody diluted in PBS with BSA 0.1% before 30 min of incubation with peroxidase-conjugated streptavidin. For revelation, Novared revelation kit (SK-4800, Vector) was used and tissues were counterstained with Mayer’s hematoxylin. Tissue were then dehydrated and slides were mounted using Cytoseal^TM^ 60. Slides were scanned with Zeiss AxioScan Z1 (Carl Zeiss) and H3ac-positive (staining, brown nuclei) and H3ac-negative (unstained, blue nuclei) cells were counted manually. For HDAC5 staining, the intensity of the signal was rated as following: 0- no signal, 1- low intensity signal, 2- high intensity signal, 3- very strong intensity signal (**Figure S7**).

#### Microarray experiments for measurement of gene expression in ileal mucosa of CD patients

Total RNA was extracted using TRizol reagent and purified using RNeasy micro kit (Qiagen) according to the manufacturer’s instructions. Using a NanoDrop 2000 Spectrophotometer (ThermoFisher Scientific), absorbance at 260 nm and 280 nm was measured to determine the quantity and purity of total extracted RNA. Quality of samples was then checked with the Caliper LabChip GX High-Throughput Bioanalyzer (Life Sciences). Microarray data were generated using the Affymetrix GeneChip Human Genome U133 Plus 2.0 Arrays, which comprise 54 675 probe sets as described.^42^ The microarray raw data were preprocessed to obtain log2 expression value and normalized using robust multi-array average (RMA) method as implemented in the affy R package. Batch effect removal was performed using SVA R package to correct for technical batch effects.

### Western blot and antibodies

Proteins of treated Caco-2 cells were extracted with the addition of 300 µL/well of lysis buffer (60 mM Tris HCl pH 6.8, SDS 2% (v/v), glycerol 10%, DTT (w/v) 0.3%, bromophenol blue). Whole cell extracts were sonicated (ON: 15s; OFF: 15s; 3 cycles) and proteins were boiled 10 min at 98°C. For colonic protein, one centimeter of mucosa was extracted and suspended in cell lysis buffer (60 mM Tris HCl pH 6.8, SDS 2% (v/v), glycerol 10%) supplemented with protease inhibitor (cOmplete^TM^ Protease Inhibitor Cocktail, Roche). Whole cell extracts were sonicated as previously and Laemmli buffer was added to proteins (1:4) before boiling (5 min at 98°C).

Proteins were loaded in a 12% SDS-PAGE gel for electrophoresis migration and were transferred to a nitrocellulose membrane (Trans-Blot® Transfer System, Bio-Rad). After 1 h of incubation in blocking buffer (PBS-Tween 20 0.05%, 5% BSA), membranes were blotted with the primary antibodies anti-H3K9ac, anti-H3ac (1/3000, #61251, #61637, Active Motif), anti-HDAC1, anti-HDAC2, anti-HDAC3, anti-HDAC4, anti-HDAC6, anti-HDAC7 (1/2000, #5356, #2540, #3949, #15164, #7612, #33418, Cell Signaling Technologies), anti-HDAC5 (1/2000, #20458, Cell Signaling Technologies (only in **Figure S2**) or 1/500, #sc-133106, Santa Cruz Biotechnology), anti-HDAC8 (1/2000, #ab187139, Abcam) and anti-GAPDH (1/5000, #5174, Cell Signaling Technologies) overnight at 4°C. Membranes were washed 3 times with PBS-Tween 20 0.05% and then incubated with appropriate HRP-conjugated secondary antibodies diluted in blocking buffer (1/10000) for 1 h at room temperature. Membranes were washed 3 times and proteins were detected using ECL substrate (Enhanced ChemiLuminescence, Clarity^TM^ Western ECL Substrate, Bio-Rad). Bands intensities were quantified using Image Lab software (Bio-Rad).

### Statistics

Values are expressed as the mean ± SD of ‘n’ number of experiments or median. Statistical analysis was performed using GraphPad Prism 6 for Windows version 6.07 (GraphPad Software, San Diego, CA, USA) software package for PC. Unpaired Mann-Whitney test was realized for single comparisons. Ordinary one-way ANOVA corrected with Tukey’s test was performed for multiple comparisons. A value of p < .05 was considered as statistically significant. Spearman test was used for correlations analysis.

### Study approval

Animal protocols were approved by the Committee for Research and Ethical Issues of the C2E2A (“Comité d’éthique pour l’expérimentation animale Auvergne” N°002, APAFIS#18723-2019013021542445 and #33490-2021101815102774). All experiments were carried out in accordance with relevant guidelines and regulations.

The study was performed in accordance with the Declaration of Helsinki, Good Clinical Practice and applicable regulatory requirements including patient informed consent. The human study was approved by the local Ethics Committees “Comité de protection des personnes Île-de-France IV-CPP 2009/17”.

## Supplementary Material

Supplemental Material

## Data Availability

Microarrays raw data used in this study are from Ngollo et al^42^ and have been deposited in the National Center for Biotechnology Information (NCBI) Gene Expression Omnibus (GEO) and are accessible through GEO accession number (GSE186582 - Expression data from intestinal mucosa of patients with Crohn disease). https://www.ncbi.nlm.nih.gov/geo/query/acc.cgi?acc=GSE186582
