# Supplementary material for: Epigenetic master regulators HDAC1 and HDAC5 control pathobiont Enterobacteria colonization in ileal mucosa of Crohn’s disease patients
Source: Gut Microbes. 2022 Sep 29;14(1):2127444. doi: 10.1080/19490976.2022.2127444 (PMC9542275; doi:10.1080/19490976.2022.2127444)
Supplement: Supplemental Material [file KGMI_A_2127444_SM5432.pdf]

Supplementary Table S1: Characteristics of the REMIND cohort used in this study.

**n = 171 patients**		**CD patients non-colonized by Enterobacteria (n=95)**	**CD patients colonized by MAEC Enterobacteria (n=51)**	**CD patients colonized by AIEC Enterobacteria (n=25)**
Age at the time of surgery (mean ± SD)		32.8 ± 11.8	36.6 ± 12.5	37.6 ± 14.5 years
Disease duration (median, IQR)		4 [1.0-10.0] years	8 [2.5-15.0] years	5 [2.3-8.0] years
Female gender n (%)		51 (53.7%)	22 (43.1%)	13 (52.0%)
**Montreal classification**				
Location				
	L1 n (%)	55 (57.9%)	35 (68.6%)	15 (60.0%)
	L2 n (%)	0 (0%)	1 (2.0%)	0 (0%)
	L3 n (%)	38 (40.0%)	15 (29.4%)	10 (40.0%)
	L4 n (%)	2 (2.1%)	0 (0%)	0 (0%)
	Perianal lesions n (%)	20 (21.1%)	12 (23.5%)	4 (16.0%)
Behaviour				
	B1 n (%)	21 (22.1%)	5 (9.8%)	4 (16.0%)
	B2 n (%)	38 (40.0%)	27 (52.9%)	17 (68.0%)
	B3 n (%)	36 (37.9%)	19 (37.3%)	4 (16.0%)
	Prior intestinal resection n (%)	16 (16.8%)	11 (21.6%)	7 (28.0%)
**Medications before surgery**				
	No treatment n (%)	14 (14.7%)	8 (15.7%)	6 (24.0%)
	Antibiotics use within 4 weeks before surgery n (%)	38 (40.0%)	12 (23.5%)	7 (28.0%)
within 3 months before surgery	Corticothérapie n (%)	29 (30.5%)	20 (39.2%)	6 (24.0%)
	Azathioprine n (%)	19 (20.0%)	24 (47.1%)	3 (12.0%)
	Methotrexate n (%)	2 (2.1%)	2 (3.9%)	1 (4.0%)
	Anti-TNF agents n (%)	46 (48.4%)	27 (52.9%)	15 (60.0%)

SD: standard deviation, IQR: interquartile range, n: number, TNF: Tumor Necrosis Factor

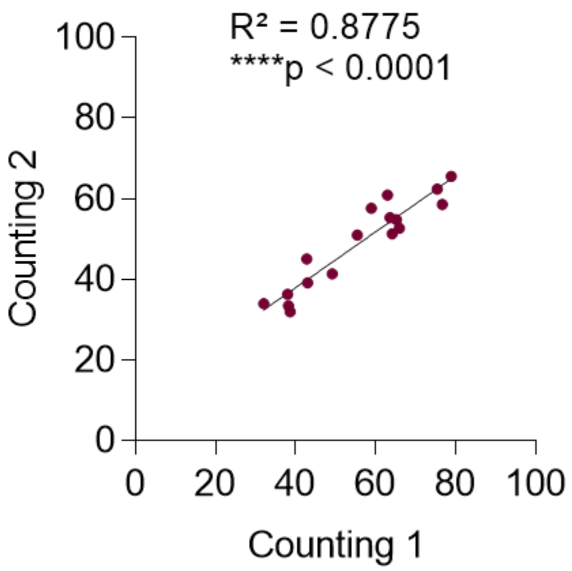

Figure S1: Validation of the counting method. Correlation between the results of the two countings of H3ac positive cells made by 2 different persons (n=17). Correlation existing between two variables were assessed by a Spearman test.

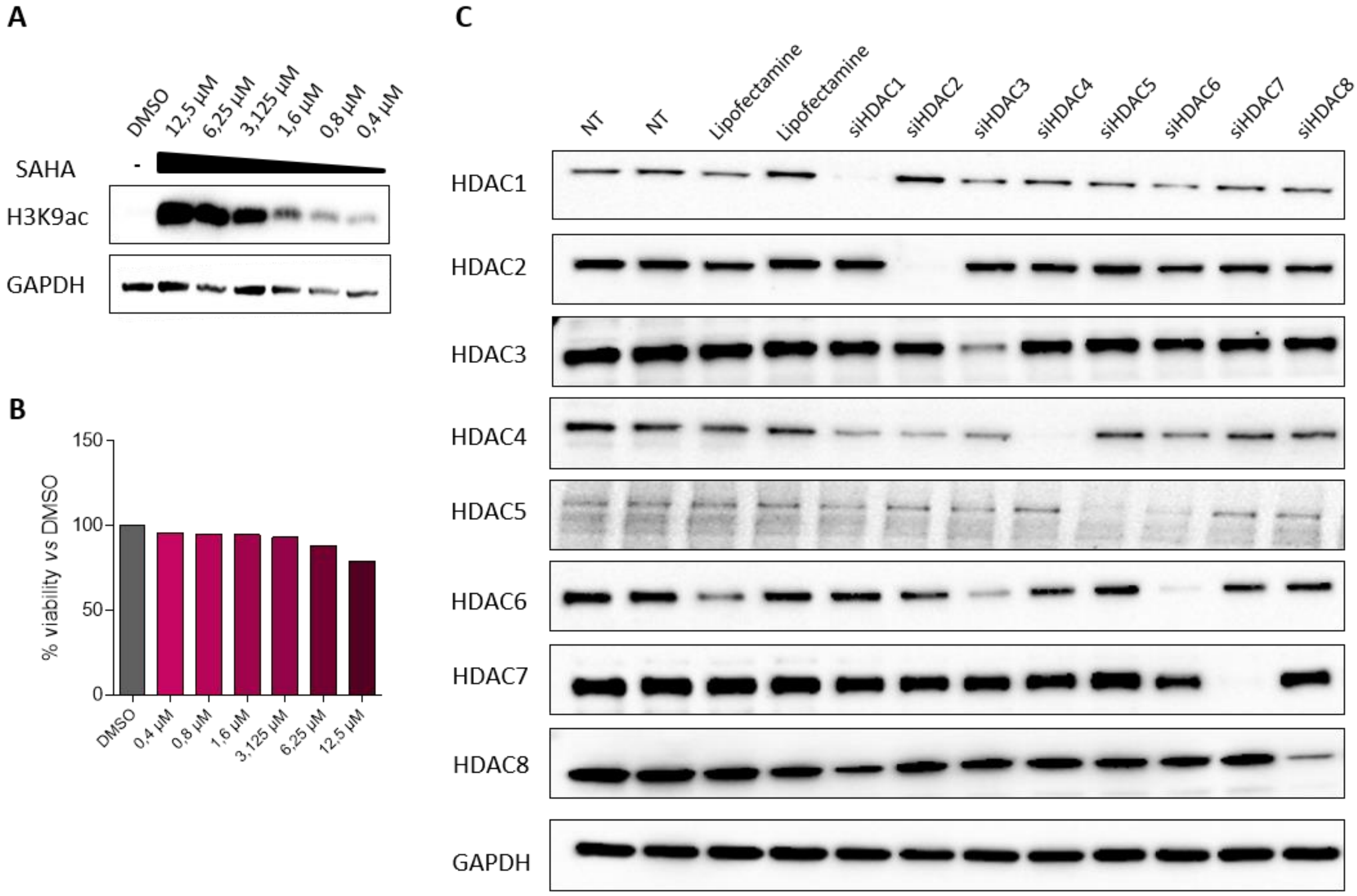

Figure S2: Validation of the efficacy of the global HDAC inhibitor SAHA and of siRNAs. A: Western blot targeting H3K9ac mark performed on Caco-2 cells treated with different concentrations of HDAC inhibitor SAHA. **B**: Cell viability was determined after 24h of SAHA treatment (n=1). **C**: Western blot performed on cells transfected with siRNA targeting the different HDAC. NT: untreated.

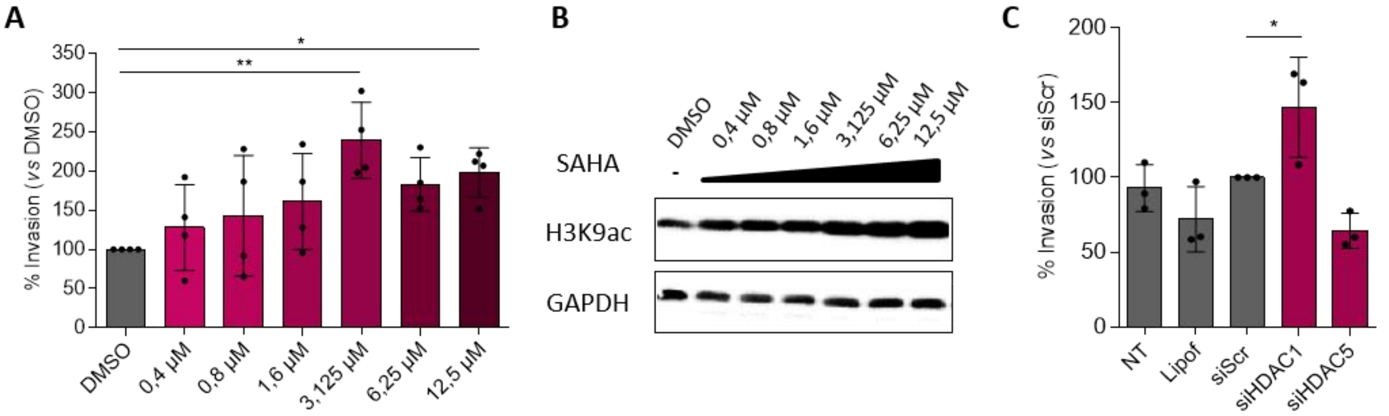

Figure S3: Validation of the effect of the global HDAC inhibitor SAHA and siRNAs on T84 cell line A: T84 cells were treated with increasing concentrations of HDAC inhibitor SAHA for 24h before infection with the AIEC strain LF82 at MOI 100. A gentamicin protection assay was performed to evaluate invasive ability of the strain in the different conditions (n=4). **B**: Western blot targeting H3K9ac mark performed on T84 cells treated with different concentrations of HDAC inhibitor SAHA. **C**: T84 cells transfected with control siRNA (siScr) or siRNA directed against HDAC1 and HDAC5 were infected with AIEC strain LF82 at MOI 100. Invasive bacteria were numbered at 4h post-infection (n=3). The results are the mean ± SD. One-way ANOVA, *p<0.05, **p<0.01.

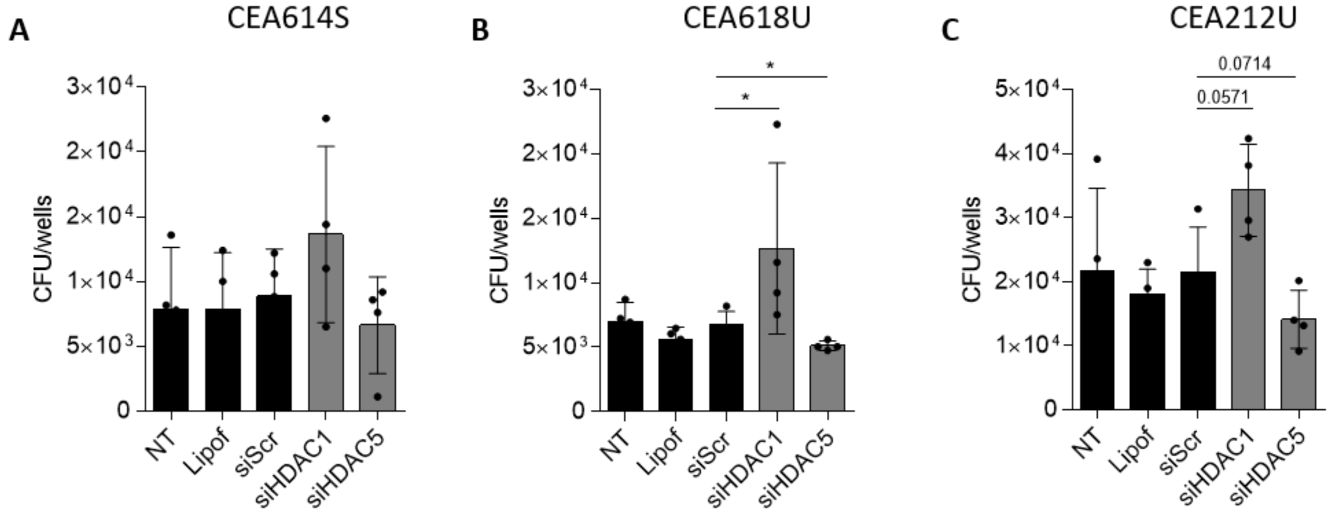

Figure S4: HDAC1 and HDAC5 inversely regulate the invasive ability of different AIEC strains. Caco-2 cells transfected with siRNA directed against HDAC1 and HDAC5 were infected with 3 different AIEC strains: CEA614S (**A**), CEA618U (**B**) and CEA212U (**C**) at MOI 100. A gentamicin protection assay was performed to measure the invasive ability of the strains in the different knocked-down conditions (n=4). The results are the mean ± SD. Mann-Whitney test, *p<0.05. MOI: multiplicity of infection, Lipof: lipofectamine, NT: untreated, Scr: scramble.

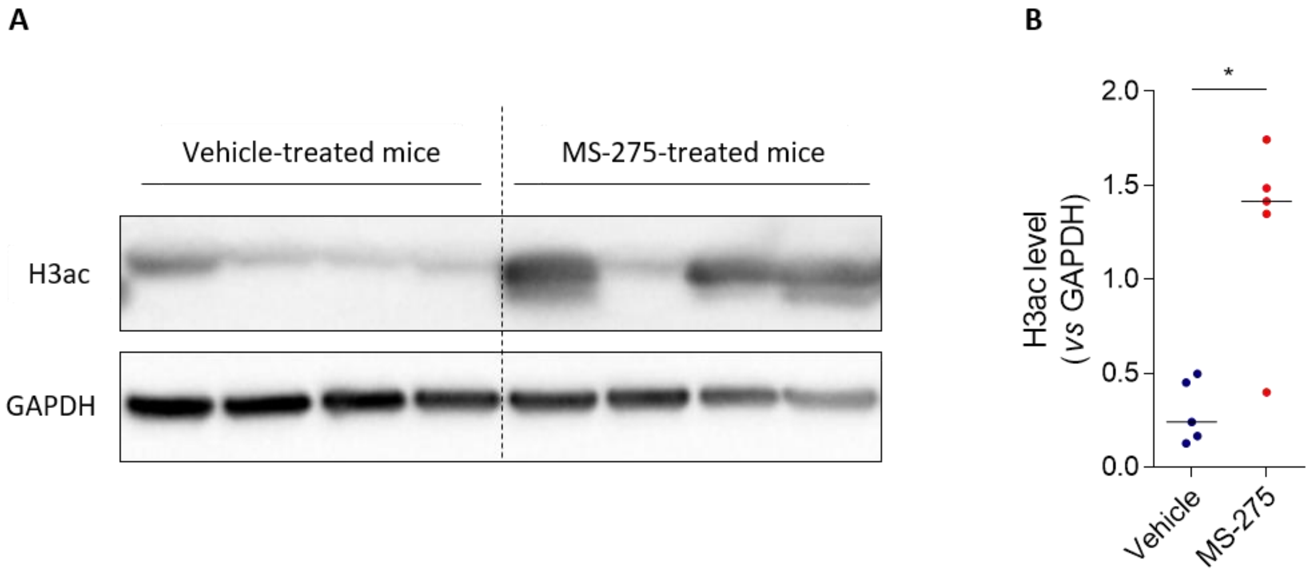

Figure S5: Validation of the efficacy of the class I HDAC inhibitor MS-275 *in vivo*. A: Western blot targeting H3ac mark performed on colonic mucosa from mice treated with 20 mg/kg/day of MS-275 for 5 days. **B**: Quantification was performed by assessing band intensities using the Image Lab software (n= 5 mice/per group). Bars represent medians. Mann-Whitney test, *p<0.05.

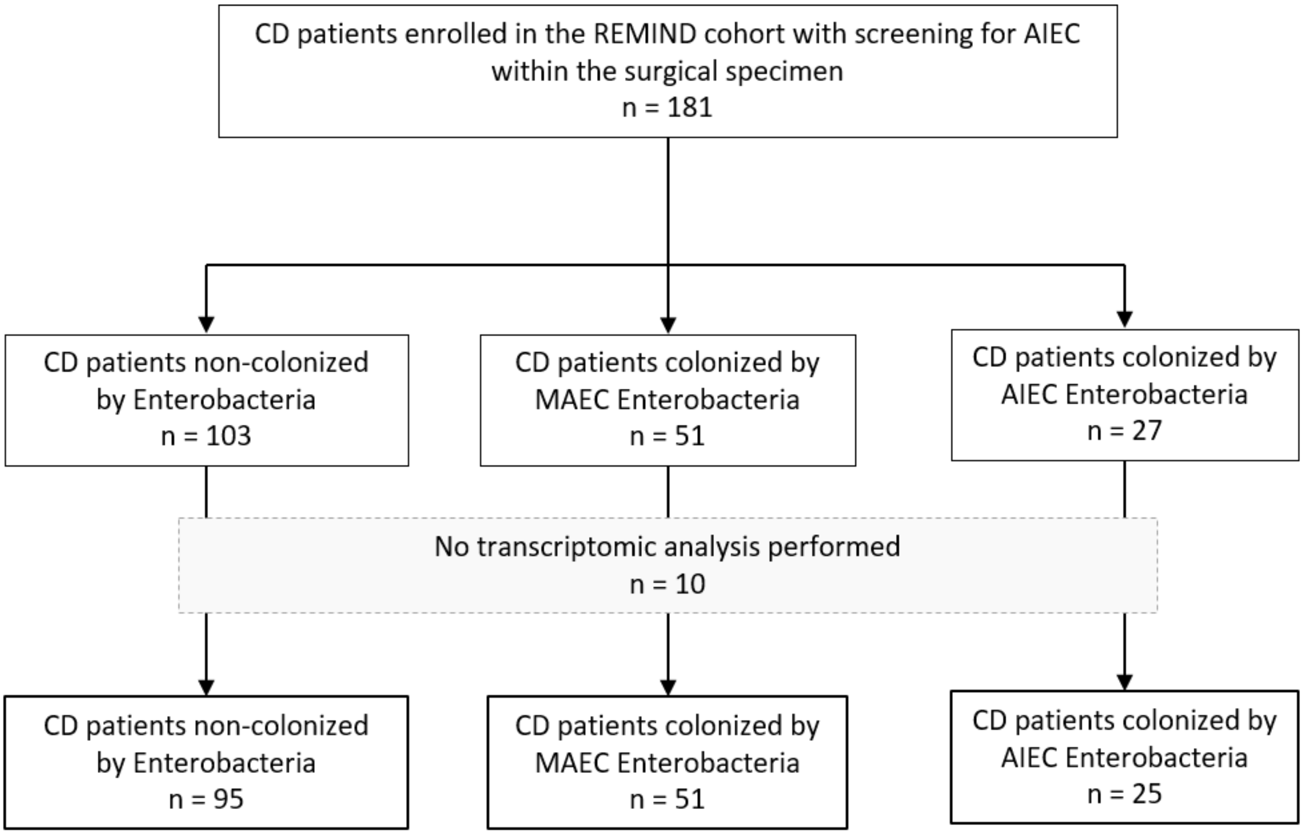

Figure S6: Presentation of the REMIND cohort. Patients for which Enterobacteria numbering and AIEC screening within the surgical specimen and transcriptomic analysis were performed were retained for the analysis (n=171). MAEC: Mucosa-associated *E. coli*.

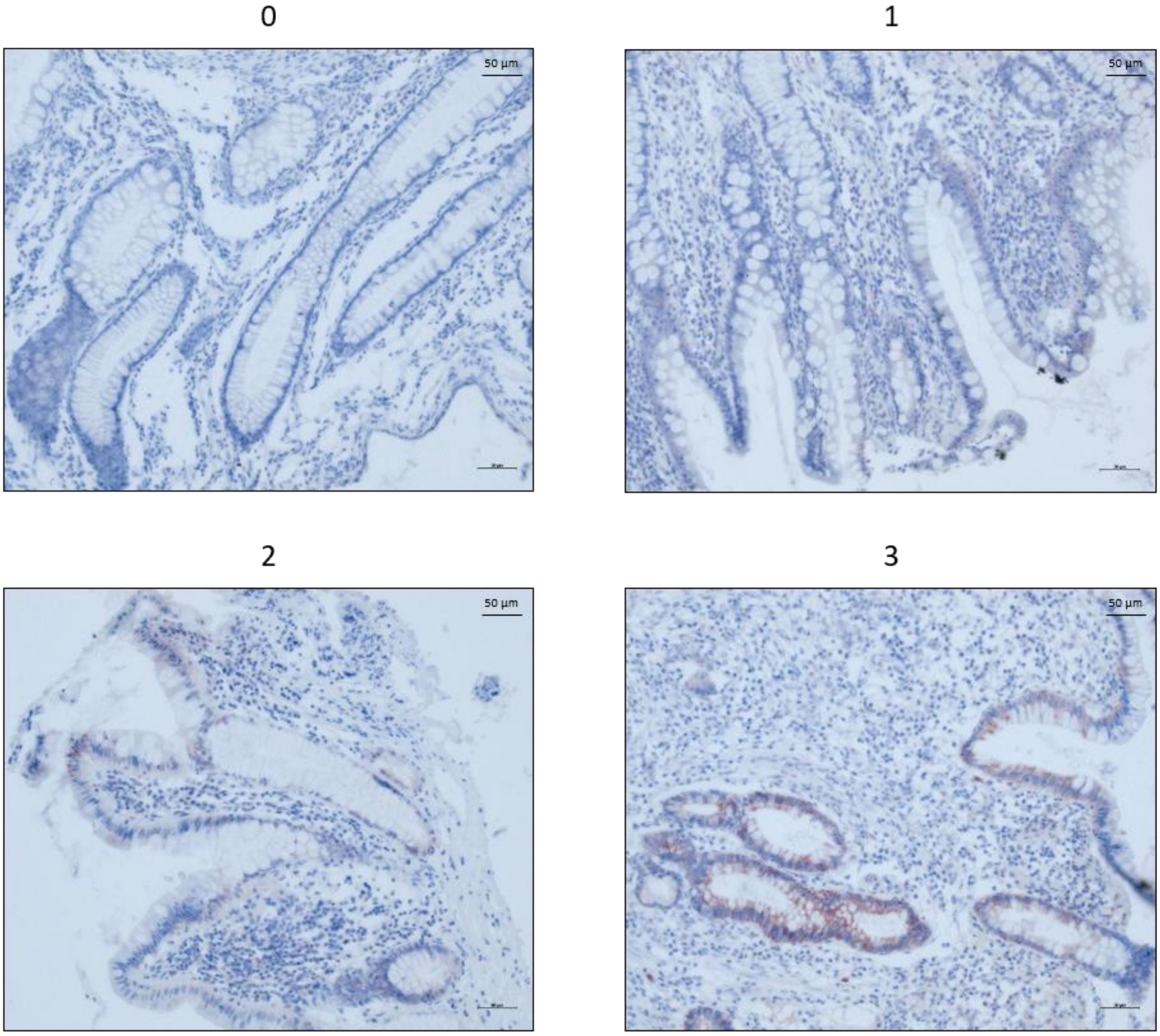

Figure S7: Scoring examples of immunohistochemical staining of HDAC5 on mucosa samples from CD patients. The intensity of the signal in samples was rated as following: 0- no signal, 1- low intensity signal, 2- high intensity signal, 3- very strong intensity signal.

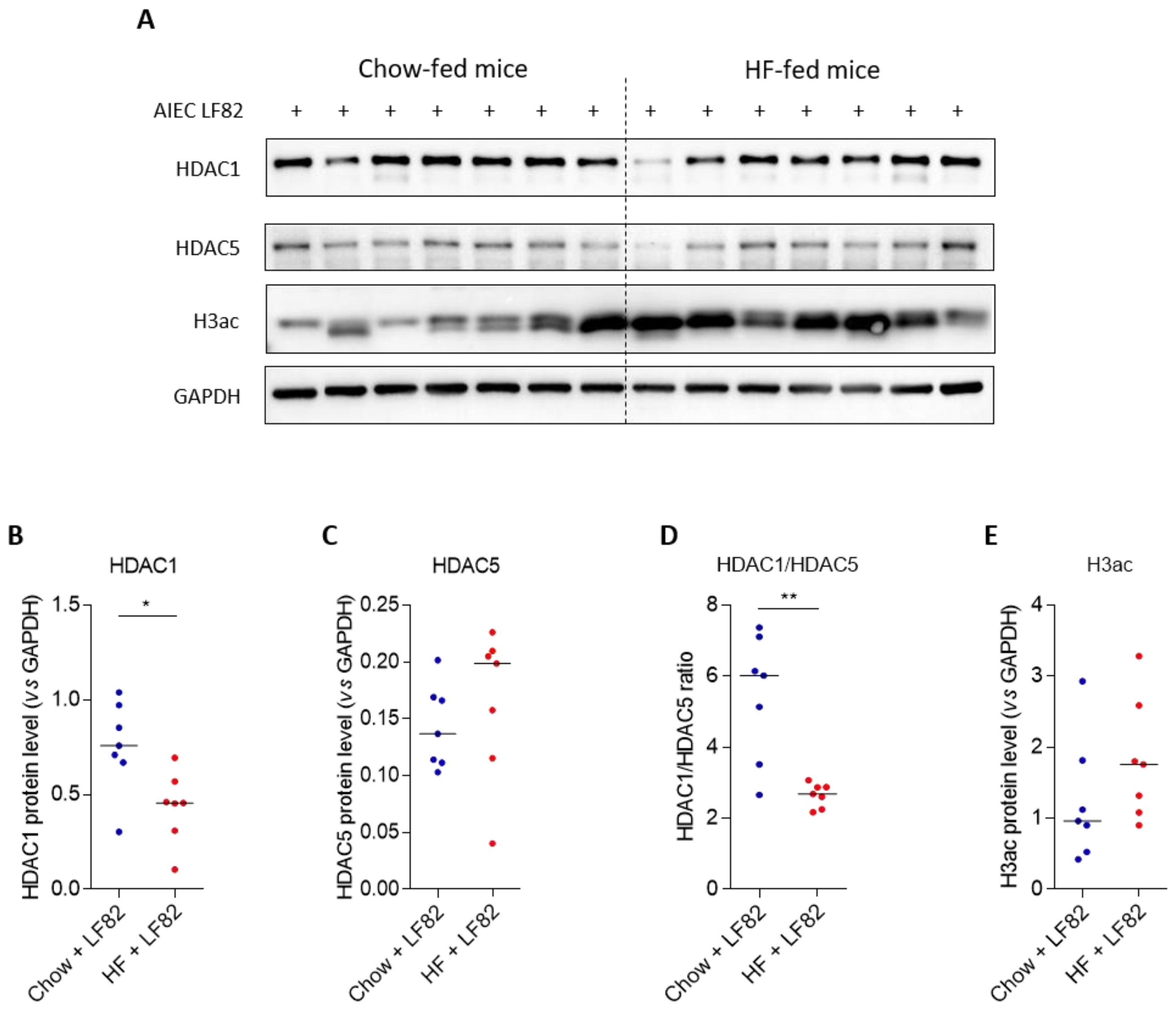

Figure S8: HF diet is required for AIEC to induce epigenetic alterations. A: HDAC1, HDAC5 and H3ac expression in colonic mucosa of Chow-fed or HF-fed mice infected with AIEC strain LF82 analyzed by western blot. **B-E:** Quantification of protein expression was performed by assessing band intensities using the Image Lab software (n= 7 mice/per group). Bars represent medians. Mann-Whitney test, *p<0.05, **p<0.01. H3ac: acetylated H3.
